# Could [^18^F]FDG PET/CT or PET/MRI Be Useful in Patients with Skull Base Osteomyelitis?

**DOI:** 10.3390/diagnostics12092035

**Published:** 2022-08-23

**Authors:** Francesco Dondi, Domenico Albano, Giorgio Treglia, Francesco Bertagna

**Affiliations:** 1 Nuclear Medicine, ASST Spedali Civili di Brescia, 25123 Brescia, Italy; 2 Nuclear Medicine, Università degli Studi di Brescia and ASST Spedali Civili di Brescia, 25123 Brescia, Italy; 3 Clinic of Nuclear Medicine, Imaging Institute of Southern Switzerland, Ente Ospedaliero Cantonale, 6500 Bellinzona, Switzerland; 4 Department of Nuclear Medicine and Molecular Imaging, Lausanne University Hospital and University of Lausanne, 1015 Lausanne, Switzerland; 5 Faculty of Biomedical Sciences, Università della Svizzera Italiana, 6900 Lugano, Switzerland

Skull base osteomyelitis (SBO) is an uncommon infection that can have a devastating impact on patient survival if not timely recognized and treated [1]. Usually, SBO develops as a worsening consequence of otologic or sinogenic infections in older, immunosuppressed or diabetic patients [1,2]. Symptoms of SBO can be subtle and non-specific, such as the presence of persistent headache, otalgia or otorrhea [3,4]. Diagnosis of SBO strongly relies on imaging techniques such as magnetic resonance imaging (MRI), able to evaluate soft tissues involvement, and computed tomography (CT) that can assess the bone status [3].

^18^F-fluorodeoxyglucose ([^18^F]FDG) is the most common radiotracer used to perform positron emission tomography/CT (PET/CT) and PET/MRI. At first, this tracer was used mainly for the assessment of neoplastic diseases, given its ability to detect hypermetabolic lesions on the basis of their glycolytic metabolism [5,6,7,8,9,10,11]. However, high glucose metabolism characterizes also activated inflammatory cells, and the use of [^18^F]FDG PET has emerged for the evaluation of infectious and inflammatory diseases [12,13,14,15,16,17,18]. In this setting, the role of [^18^F]FDG PET imaging for the assessment of SBO is emerging.

In general, [^18^F]FDG PET/CT has demonstrated high sensitivity and specificity for the diagnosis of SBO. In particular, its sensitivity, specificity, positive predictive value, negative predictive value and accuracy in the diagnosis of SBO were reported as 96.7%, 93.3%, 98.3%, 87.5% and 96.1%, respectively, with an accuracy comparable with that of MRI [1,19]. Interestingly, a significant agreement between PET/CT and MRI for the assessment of both bone and soft tissue involvement was found [19]. Both [^18^F]FDG PET/CT or PET/MRI are able to visualize increased radiopharmaceutical uptake on the bone and the surrounding soft tissues involved by the infectious process [2,4,20,21,22,23,24,25].

The role of [^18^F]FDG PET/CT for the assessment of response to treatment in SBO has also been investigated with encouraging results [1,3,19,20,21,25,26]. In this setting, it has been reported the concordance between the resolution of abnormal uptakes at [^18^F]FDG PET with the clinical presentation of the patients. Furthermore, the ability of [^18^F]FDG PET/CT to allow the discontinuation of therapy in SBO has also emerged [20]. It has been reported that patients that did not demonstrate any increased metabolic activity at the time of [^18^F]FDG PET/CT evaluation remained free of symptoms; conversely, it was suggested to continue the treatment of SBO until the normalization of [^18^F]FDG uptake, also in asymptomatic patients [27]. Interestingly, if performed in early stages of SBO, [^18^F]FDG PET/CT may also allow the early identification of the most suitable site to perform biopsy, in order to establish a clear microbiological diagnosis and therefore start a specific therapy [20]. An excellent correlation between inflammatory markers and [^18^F]FDG PET/CT was also reported and concordance of C-reactive protein (CRP) levels with PET/CT results and their ability to guide the follow-up of patients were underlined in some cases [21,27].

The nature of the pathogens involved in SBO can influence the results of [^18^F]FDG PET/CT imaging. In this setting, it was reported that in patients with fungal infections low-grade [^18^F]FDG uptake or false negative findings can be present on [^18^F]FDG PET/CT [1]. Furthermore, significantly higher [^18^F]FDG uptake for bacterial and mixed organism infections compared to fungal infections were reported [19]. [^18^F]FDG uptake was reported as an independent prognostic factor for SBO also compared to serum inflammatory markers [19].

[^18^F]FDG PET/MRI has been recently used for the assessment of SBO, given its ability to combine the soft tissue contrast of MRI with the metabolic evaluation of PET. Since this imaging method combines functional and high-resolution morphological images, it gives a more accurate measure about the inflammatory process [3]. The MRI component is able to reveal hyperintensities in T1-weighted images suggestive for SBO, corresponding to areas of increased [^18^F]FDG uptake on the PET component. Interestingly, [^18^F]FDG PET/MRI conjugates the complementary values of PET and MRI for the characterization of cell metabolism and the tissue microenvironment in SBO [23]. Results on [^18^F]FDG PET/MRI in SBO are promising and it has been suggested to perform both the initial work-up and the follow-up of SBO with [^18^F]FDG PET/MRI, reserving [^18^F]FDG PET/CT as the second best option in cases of contra-indications to perform MRI [3].

Conventional nuclear medicine imaging techniques have also been used for decades in SBO evaluation [1,28]. In this setting, this disease can be assessed with radiolabeled leukocyte scintigraphy (LS). A comparison between such imaging modality and [^18^F]FDG PET/CT has been performed, reporting a sensitivity and a specificity of 86% and 75% for LS, and of 43% and 100% for [^18^F]FDG PET/CT in healing assessment setting [28].

Recently, PET/CT with [^18^F]FDG labeled autologous leukocytes has been used for the diagnosis of SBO, with promising results given the low radiopharmaceutical uptake in the brain on this imaging modality and the high resolution of PET/CT imaging [24,29].

[^18^F]FDG PET/CT was also used for the assessment of necrotizing external otitis that, by definition, consists of osteomyelitis of the temporal bone. In this scenario, hybrid imaging was suggested as a reliable modality for the diagnosis, the disease localization and the decision-making regarding treatment cessation [30].

In conclusion, taking into account literature data, we believe that [^18^F]FDG PET/CT or PET/MRI should be considered as useful and reliable tools for the assessment of SBO, both at diagnosis and for the assessment of response to treatment, even if more studies in these settings are warranted.
